# Impact of Software Settings on Multiple-Breath Washout Outcomes

**DOI:** 10.1371/journal.pone.0132250

**Published:** 2015-07-13

**Authors:** Selina Summermatter, Florian Singer, Philipp Latzin, Sophie Yammine

**Affiliations:** 1 University Children’s Hospital Basel, UKBB, 4031, Basel, Switzerland; 2 University Children’s Hospital Bern, 3010, Bern, Switzerland; 3 University Children’s Hospital Zurich, 8032, Zurich, Switzerland; University of Tübingen, GERMANY

## Abstract

**Background and Objectives:**

Multiple-breath washout (MBW) is an attractive test to assess ventilation inhomogeneity, a marker of peripheral lung disease. Standardization of MBW is hampered as little data exists on possible measurement bias. We aimed to identify potential sources of measurement bias based on MBW software settings.

**Methods:**

We used unprocessed data from nitrogen (N_2_) MBW (Exhalyzer D, Eco Medics AG) applied in 30 children aged 5–18 years: 10 with CF, 10 formerly preterm, and 10 healthy controls. This setup calculates the tracer gas N_2_ mainly from measured O_2_ and CO_2_concentrations. The following software settings for MBW signal processing were changed by at least 5 units or >10% in both directions or completely switched off: (i) environmental conditions, (ii) apparatus dead space, (iii) O_2_ and CO_2_ signal correction, and (iv) signal alignment (delay time). Primary outcome was the change in lung clearance index (LCI) compared to LCI calculated with the settings as recommended. A change in LCI exceeding 10% was considered relevant.

**Results:**

Changes in both environmental and dead space settings resulted in uniform but modest LCI changes and exceeded >10% in only two measurements. Changes in signal alignment and O_2_ signal correction had the most relevant impact on LCI. Decrease of O_2_ delay time by 40 ms (7%) lead to a mean LCI increase of 12%, with >10% LCI change in 60% of the children. Increase of O_2_ delay time by 40 ms resulted in mean LCI decrease of 9% with LCI changing >10% in 43% of the children.

**Conclusions:**

Accurate LCI results depend crucially on signal processing settings in MBW software. Especially correct signal delay times are possible sources of incorrect LCI measurements. Algorithms of signal processing and signal alignment should thus be optimized to avoid susceptibility of MBW measurements to this significant measurement bias.

## Introduction

Assessment of impaired ventilation distribution in the lungs by multiple-breath washout (MBW) measurement has been increasingly used over the past few years. In children with cystic fibrosis (CF) but also with primary ciliary dyskinesia MBW has been shown to be more sensitive for detection of early structural lung changes than standard lung function tests [1–3]. MBW is able to assess treatment effects even in mild CF lung disease [4–6]. In some centers, MBW is already part of the routine clinical surveillance in patients with CF [7–9].

The recently published ATS/ERS consensus aims to standardize MBW signal recording, processing, and analysis [10]. Despite guidelines as to what software packages should be able to perform, most of the technical recommendations rely on little data only [11;12]. In particular, uncertainty exists as to what extent the different software settings impact upon MBW outcomes. Even if the MBW post-hoc quality criteria are met [10], technical flaws during or after the measurement may strongly impact on MBW results. Random measurement bias may particularly influence longitudinal MBW data. Further, some MBW data require post hoc offline analysis to adjust for incorrect settings of online measurements. This may introduce additional non-systematic bias on test results.

To avoid MBW measurement bias as good as possible, the most important software settings and the respective impact upon results need to be known. The aim of our study was thus to assess the influence of different software settings on MBW results. We used nitrogen (N_2_) MBW raw data from 30 children and adolescents with and without lung disease. We systematically evaluated the impact of changing different software parameters in order to identify the most important source of measurement bias. Primary outcome was the change in lung clearance index (LCI) and functional residual capacity (FRC), secondary outcome was change in phase III slope indices (SIII).

## Methods

### Subjects

To cover a wide age and disease range we used unprocessed raw N_2_MBW data (A-files from the recording software Spiroware 3.1.6, Eco Medics AG, Duernten, Switzerland) from children aged 5 to 18 years, 10 with CF, 10 formerly preterm born and 10 healthy term born children. The study was approved by the Ethics Committee of the Canton of Bern, Switzerland. The children’s assent was obtained and parents or caregivers provided written informed consent.

### Nitrogen multiple-breath washout

**Fig 1:**
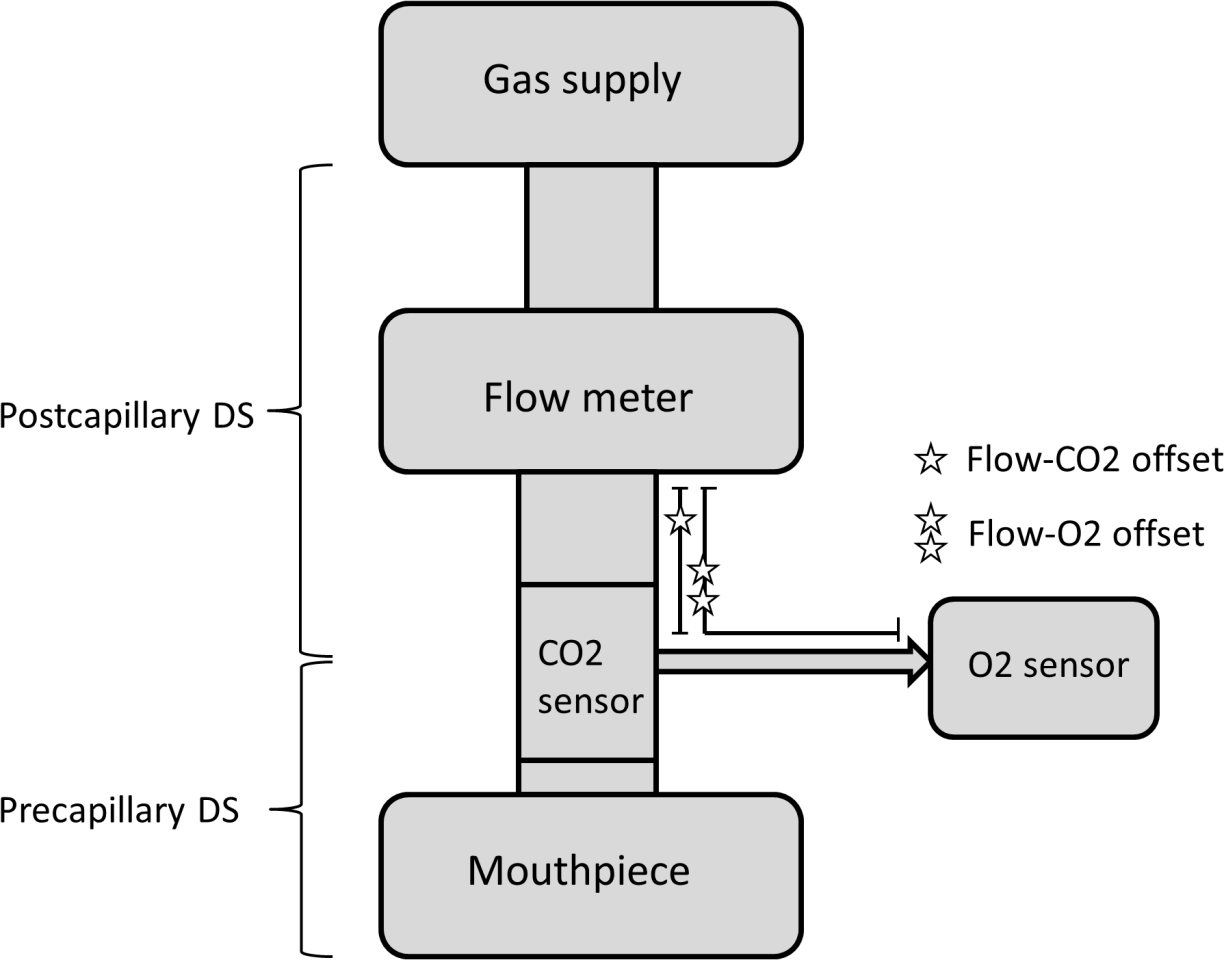
Schematic design of the N_2_MBW setup. The figure illustrates the patient interface and measurement points of the current nitrogen multiple-breath washout (N_**2**_MBW) setup device (Exhalyzer D). Flow (and derived volume) are measured by a mainstream ultrasonic flowmeter. Gas concentrations are measured by the side-stream laser O_**2**_ sensor and the main-stream infra-red CO_**2**_ sensor. The N_**2**_ fraction is measured indirectly by F_**N2**_ = 1 –F_**O2**_ –F_**CO2**_ –F_**Argon**_. The gas sampling port divides pre- from post-capillary dead space. Star symbols give approximates of volume and thus delay times (off set) between gas and flow sampling points. The gas supply illustrates the open bypass; during the N_**2**_MBW the patient breathes 100% O_**2**_ through the mouthpiece.

We applied a previously described N_2_MBW setup [13;14] (Exhalyzer D and Spiroware 3.1.6, Eco Medics AG) as recommended by the current consensus and the manufacturer to record raw data [10]. In this device flow was measured by a mainstream ultrasonic flowmeter which derives tidal volumes (Fig 1). Gas concentrations were measured by a side-stream laser O_2_ sensor and a main-stream infra-red carbon dioxide (CO_2_) sensor. The N_2_ fraction was measured indirectly from O_2_, CO_2_ and the (estimated) Argon fraction. Flow-gas delay times were based on default settings as recommended by the manufacturer and further individually adjusted based on visual control of the N_2_ signal. Equipment dead space volume was divided in pre and post sampling-point volumes, defining pre- and post-capillary dead space as represented in Fig 1. Depending on the child’s bodyweight appropriate apparatus dead space reducer was used, set 2 (9.5mL) for children <35kg and set 3 (22mL) for children >35kg, except for the use of set 3 in two preterm children and two children with CF with <35kg bodyweight for logistic reasons.

All children performed triplicate N_2_MBW according to current consensus [10]. During measurement children were sitting upright, wearing a nose clip and quietly breathing through a snorkel mouth piece. N_2_MBW was stopped after 3 breaths below 1/40^th^ of N_2_ starting concentration. We included the first high quality measurement per child for analysis, i.e. one test without visible breathing irregularity or leak [10].

### Software settings

All data were recorded, processed and analyzed using Spiroware 3.1.6 (Eco Medics AG). To generate a consistent baseline, original measurements were analyzed with averaged delay times per set (Table 1), averaged ambient temperature and pressure. This was the standard baseline analysis to which we compared MBW results after rerunning measurements with alternative software settings. We calculated LCI and FRC as primary outcomes, and parameters calculated from SIII (Scond and Sacin) as secondary outcomes. Outcomes were calculated as currently recommended [10], LCI from cumulative expired volume used to wash out to 1/40^th^ of initial N_2_ concentration, divided by FRC.

We based the magnitude of change in the software settings either on given setting properties or on observed changes in clinical measurements. Accordingly, we inactivated certain settings (e.g. signal correction) or changed baseline setting in both directions (increase and decrease) using realistic steps of at least 5 units (e.g. 5°C ambient temperature or 5 mL dead space) or a relative change of at least 10% (e.g. flow-O_2_ offset). We categorized our changes into four groups:

#### 1. Environmental conditions

Default ambient temperature was 21°C and pressure 980 hPa. We changed temperature from 21°C to 16°C and 26°C (±5°C or ±24%), and pressure from 980 hPa to 960 hPa and 1000 hPa (±20 hPa or ± 2%). Secondly, BTPS (body temperature pressure saturation) correction was completely switched off.

#### 2. Apparatus dead space

Pre-capillary dead space was 24 mL; post-capillary dead space 9.5 mL in set 2, and 22 mL in set 3. First we altered the default pre- and post-capillary dead spaces separately. Pre-capillary dead space was changed from 24 mL to 19 mL and 29 mL (±5 mL or ±21%); post-capillary dead space from 9.5 mL to 7.5 mL and 11.5 mL (±2 mL or ±21%) in set 2, and from 22 mL to 17 mL and 27 mL (±5 mL or ±23%) in set 3. Secondly, we simultaneously lowered and elevated both pre- and post-capillary dead spaces by ±5 mL, respectively ± 2ml for post-capillary dead space in set 2.

#### 3. Signal processing and breath detection limits

We switched off one by one the default algorithms processing raw gas signals as recommended by the manufacturer and by previous work [13]: (i) We separately and individually deactivated: automated O_2_-drift correction, dynamic CO_2_-correction (adjusting the CO_2_ signal for high O_2_ fractions), O_2_ response-time correction (normally set to 30 ms), and the correction for re-inspired N_2_ volume. (ii) We decreased the sensitivity for breath detection by elevating the required minimum tidal volume from 25 mL to 100 mL. (iii) We increased the cut-offs determining limits for SIII calculation from 65%–95% to 50%–80% of expired volume. The latter limits may include phase II in paediatric tracer gas expirograms [10].

#### 4. Signal delay times

In the current setup side-stream O_2_ and main-stream CO_2_ signals must be aligned in time together with the flow signal to allow calculation of the tracer gas N_2_ volumes as illustrated in Fig 1. Default flow-O_2_ offset was 601 ms in set 2 and 618 ms in set 3, default flow-CO_2_ offset was 51 ms in set 2 and 60 ms in set 3 (Table 1). We assessed the susceptibility of measurements towards changes in delay times by the following steps. (i) First, we changed the signal delays individually using realistic steps: For flow-O_2_ offset from 601 ms in set 2 and from 618 ms in set 3 to ±20 ms (±3%), ±40 ms (±7%) and ±60 ms (±10%); for flow-CO_2_ offset from 51 ms to ±10 ms (±25%) in set 2, and from 60 ms to ±10 ms (±17%) in set 3 (Table 1). (ii) Secondly, we changed both O_2_ and CO_2_ signal delays simultaneously in four separate analyses adding or subtracting the same times as before. (iii) Third, we maintained always a constant delay difference between the two signals in three separate analyses: we changed both O_2_ and CO_2_ signal delays identically by -40 ms, +40 ms and +80 ms, respectively.

**Table 1:** Baseline settings per patient group.

	Healthy (n = 10)	Preterm (n = 10)	CF (n = 10)
**Age** (years)	6.1 ± 0.1 [6.0–6.4]	7.9 ± 0.8 [6.6–9.2]	12.3 ± 3.6 [5.1–18.1]
**Dead space reducer set**	2	3*	3*
**Individual delay times**			
O_2_ delay (ms)	601 ± 4 (599–604)	615 ± 8 (608–620)	622 ± 5 (619–626)
CO_2_ delay (ms)	51 ± 3 (49–53)	58 ± 3 (56–60)	62 ± 2 (61–64)
**Averaged delay times for baseline**			
O_2_ delay (ms)	601	618	618
CO_2_ delay (ms)	51	60	60
**Individual signal change**			
O_2_ delay (ms): ±20, 40, 60 ms	581, 561, 541 and 621, 641, 661	598, 578, 558 and 638, 658, 678	598, 578, 558 and 638, 658, 678
CO_2_ delay (ms): ±10 ms	41 and 61	50 and 70	50 and 70

Data are given as mean ± SD, [range] and (95% CI). Baseline settings per patient group. Individual delay times per file, averaged delay times per dead space reducer set used as baseline, and individual changes for O_2_ and CO_2_ delay.

*For logistic reasons at the time of measurement in two children of the preterm group and two children of the CF group we had to use set 3 instead of 2.

### Statistics

Primary outcome parameters were changes in LCI and FRC. Change in either outcome exceeding 10% was considered relevant as 10% approximately reflects between day-to-day variability of LCI using the same equipment [13;14]. We further assessed changes in parameters calculated from SIII: Scond reflecting convection-dependent ventilation inhomogeneity and Sacin reflecting diffusion-convection-dependent ventilation inhomogeneity. We report raw Scond and Sacin as well as corrected for tidal volume as currently recommended in children [15].

FRC, LCI and Scond and Sacin data were not normally distributed while differences between tests were normally distributed. Therefore data from paired tests were compared by the non-parametric Wilcoxon signed-rank tests. Agreement between settings (differences between paired tests) were assessed by Bland Altman plots [16]. P-values <0.05 were considered statistically significant. All analyses were done using Stata (Stata Statistical Software: Release 11. College Station, TX: StataCorp LP).

## Results

We analyzed 30 raw N_2_MBW files from 10 children with CF (5 boys), 10 former preterm children (6 boys) and 10 healthy children (3 boys). Mean (range) age of children with CF was 12.3 (5.1–18.1) years, 8.0 (6.9–9.2) in former preterm and 6.1 (6.0–6.4) years in healthy children (Table 1).

### Environmental conditions

**Fig 2:**
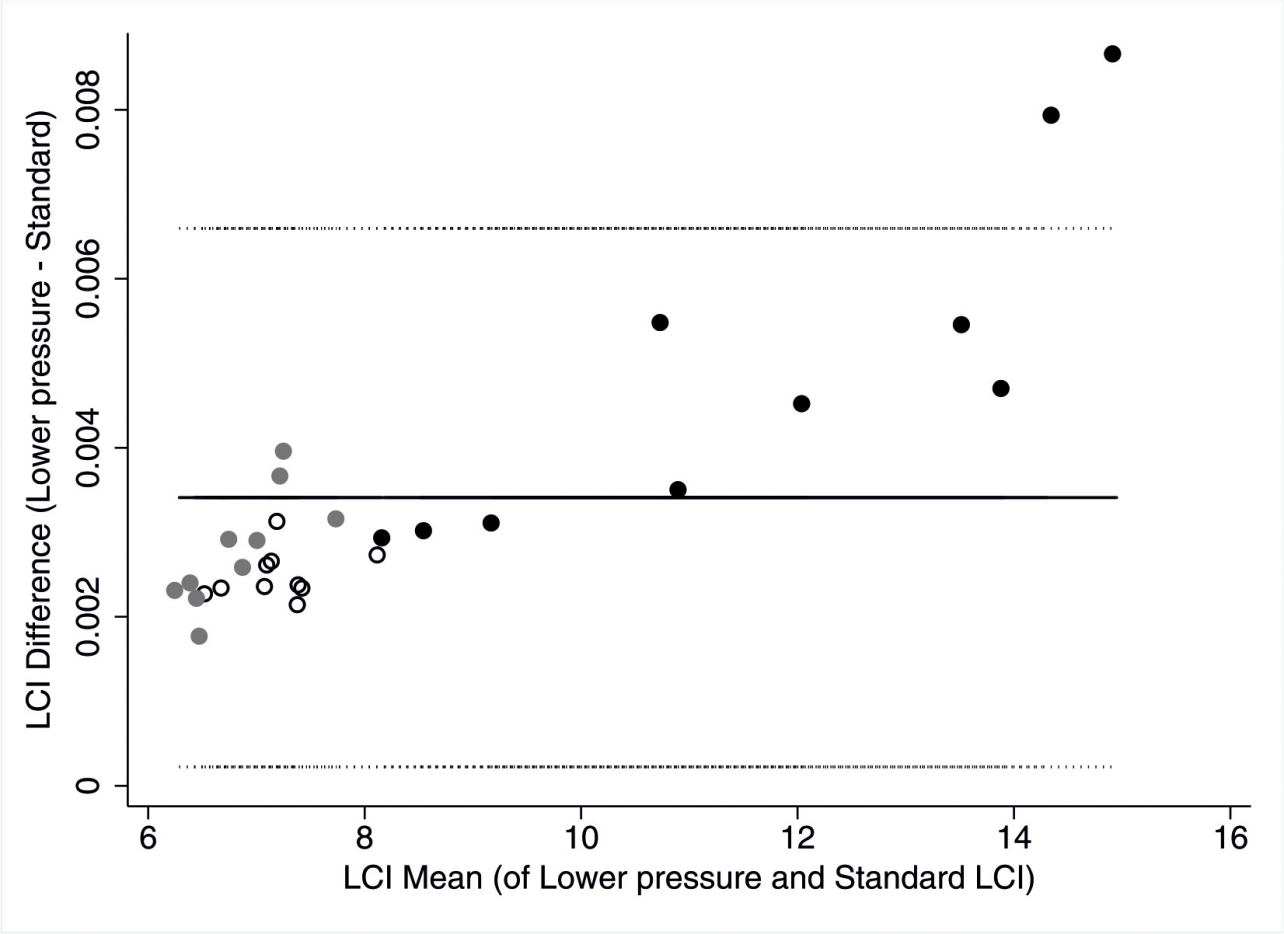
LCI measurement bias due to decreased ambient pressure settings. Bland Altmann plot of lung clearence index (LCI) determined at lower ambient pressure setting (960 kPa instead of 980 kPa) minus baseline LCI versus mean of both LCI. Black circles represent children with CF, grey circles former preterm and open circles healthy children. Solid line represent mean difference (0.0034), dotted lines represent upper (0.0066) and lower (0.0002) limits of agreement.

Temperature and pressure settings had small indirect proportional effects on FRC and LCI. Lowering the temperature by 5°C resulted in higher LCI and lower FRC values. Vice versa, 5°C higher temperature resulted in the opposite effect (Table 2). Lowering the pressure by 20 hPa lead to both higher LCI and FRC, while higher pressure lead to lower LCI and FRC (Fig 2). Effect size (mean change) of LCI in all children was lower than 10% (Table 3). Completely inactivating BTPS correction resulted in the same direction of change as lowering temperature with >10% LCI change observed in two children (one CF, one Healthy).

**Table 2:** Change of N_2_MBW results after reanalysis with different environmental conditions.

Settings	LCI	FRC	Scond	Scond*VT	Sacin	Sacin*VT
Temp. lower	↑↑	↓↓	↓	↓↓	↔	↔
Temp. higher	↓↓	↑↑	↑↑	↑↑	(↓)	↔
Pressure lower	↑↑	↑↑	↓↓	↑↑	↓↓	↓↓
Pressure higher	↓	↓	↑	↓↓	↑↑	↑↑
BTPS off	↑↑	↓↓	↔	(↓)	↑↑	(↑)

Change of N_2_MBW test results of 30 children (10 with CF, 10 former preterm and 10 healthy children) after simulation with changed environmental conditions (temperature, pressure and BTPS correction). N_2_MBW results of the standard test were compared to the test with changed setting parameter by Wilcoxon signed-rank test. We compared lung clearance index (LCI), Scond raw (Scond) and Scond corrected for tidal volume (Scond*VT), Sacin raw (Sacin) and Sacin corrected for tidal volume (Sacin*VT). The arrows reflect the statistical significance (Wilcoxon Signed Rank Test): ↓↓ difference of p < 0.001, ↓ p < 0.01, (↓) p < 0.05, ↔ no significant change.

**Table 3:** Effect size of the change in LCI after reanalysis with different environmental conditions.

Settings	Changed LCI	% change of standard LCI	# children with 5–10% change	# children with >10% change
*Baseline*	*8*.*55 ± 2*.*64* [6.29–14.94]			
Temp. lower	8.57 *±*2.64 [6.30–14.98]	0.18 *±* 0.08	0	0
Temp. higher	8.54 *±* 2.63 [6.27–14.91]	-0.17 *±* 0.07	0	0
Pressure lower	8.56 *±* 2.64 [6.29–14.95]	0.04 *±* 0.01	0	0
Pressure higher	8.59 *±* 2.66 [6.28–14.94]	0.42 *±* 1.44	1 (3%)	0
BTPS off	8.77 *±* 2.59 [6.49–15.01]	2.87 *±* 4.54	5 (16.5%)	2 (6.5%)

The table shows mean *±* SD [range] LCI after reanalysis of the tests with changed environmental conditions, mean *±* SD change in percentage of standard LCI, number of children with a change between 5–10% and children with >10% change of standard LCI.

### Apparatus dead space

**Fig 3:**
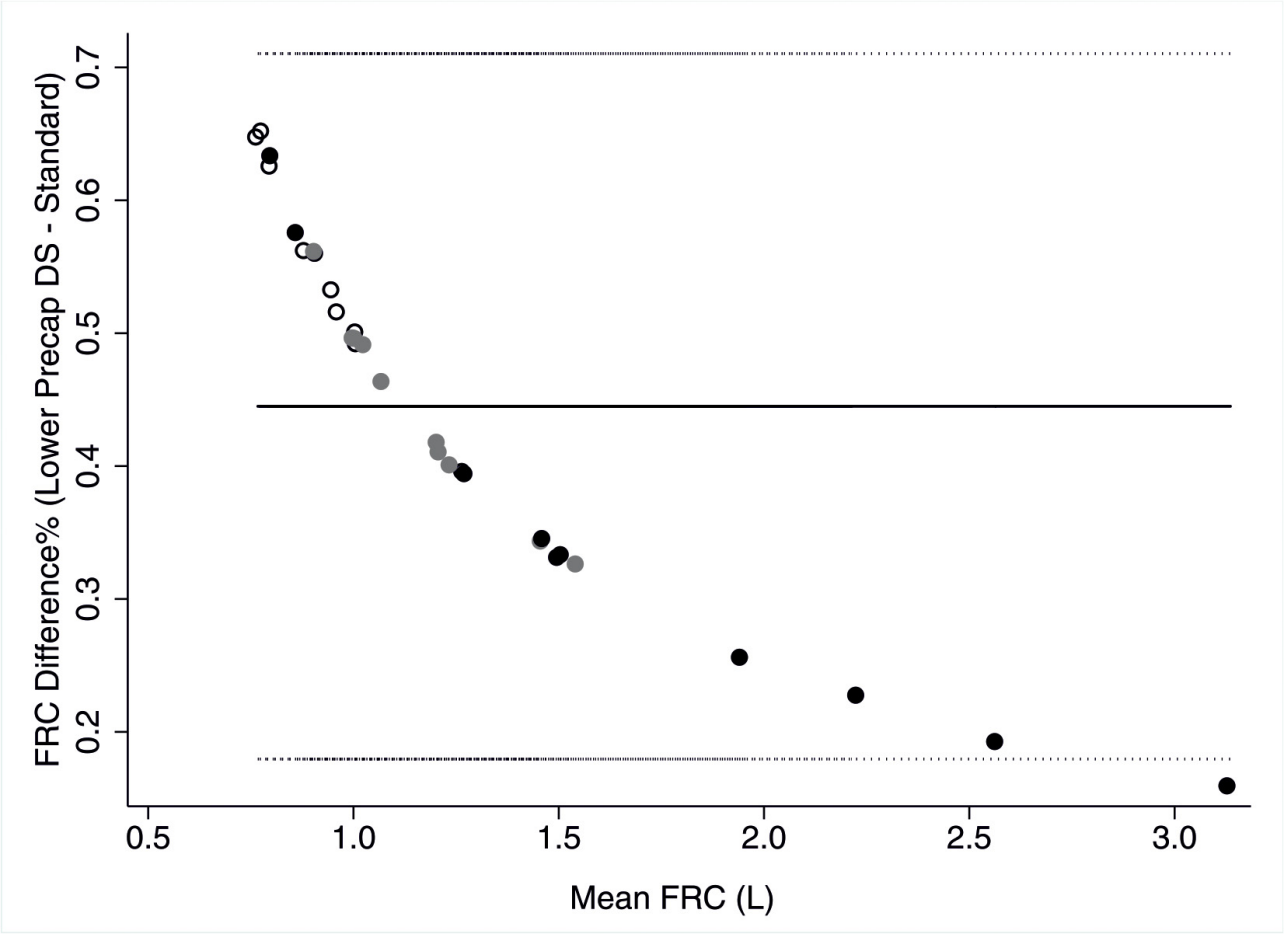
Non-linear FRC measurement bias due to decreased pre-capillary dead space. Bland Altmann plot of functional residual capacity (FRC) determined at lower pre-capillary dead space setting (19 mL instead of 24 mL) minus baseline FRC versus mean of both FRC. Black circles represent children with CF, grey circles former preterm and open circles healthy children. Solid line represent mean difference (0.45%), dotted lines represent upper (0.71%) and lower (0.18%) limits of agreement.

Dead space settings had small indirect proportional effects on FRC and LCI. Lowering pre- and post-capillary dead space settings separately and simultaneously resulted in higher LCI and FRC (Table 4). Vice versa increased dead space decreased LCI and FRC. Mean effect size on LCI was smaller than 2% and lower than 10% in all children (Tables 4 and 5). Interestingly, measurement bias due to dead space changes was strongly non-linearly associated with FRC, with smaller FRCs being most affected (Fig 3). LCI showed a trend for an analogous but much weaker relationship (data not shown).

**Table 4:** Change of N_2_MBW results after reanalysis with different apparatus dead space.

Settings	LCI	FRC	Scond	Scond*VT	Sacin	Sacin*VT
Precap DS lower	↑↑	↑↑	↓	↓	↔	↔
Precap DS higher	↓↓	↓↓	↑↑	↑↑	(↓)	(↓)
Postcap DS lower	↑↑	↑↑	↓↓	↓	↑↑	↑
Postcap DS higher	↓	(↓)	↔	(↑)	↔	↔
Pre- and postcap DS lower	↑↑	↑↑	↓	↓	↑	↑
Pre- and postcap DS higher	↓↓	↓↓	↔	↔	↔	↔

Change of N_2_MBW test results of 30 children after simulation with changed apparatus dead space (pre-capillary dead space (precap DS), post-capillary DS (postcap DS) and both). Results of standard N_2_MBW test were compared to the test with changed setting parameter by Wilcoxon signed-rank test and marked as follows: ↓↓ difference of p < 0.001, ↓ p < 0.01, (↓)p < 0.05, ↔ no significant change.

**Table 5:** Effect size of the change in LCI after reanalysis with different apparatus dead space.

Settings	Changed LCI	% change of standard LCI	# children with 5–10% change	# children with >10% change
*Baseline*	*8*.*55 ± 2*.*64* [6.29–14.94]			
Precap DS lower	8.64 *±* 2.63 [6.37–15.07]	1.13 *±* 0.57	0	0
Precap DS higher	8.46 *±* 2.64 [6.20–14.82]	-1.14 *±* 0.58	0	0
Postcap DS lower	8.61 *±* 2.65 [6.36–15.16]	0.70 *±* 0.69	0	0
Postcap DS higher	8.52 *±* 2.64 [6.20–14.69]	-0.40 *±* 1.48	0	0
Pre- and postcap DS lower	8.70 *±* 2.65 [6.44–15.28]	1.82 *±* 0.96	0	0
Pre- and postcap DS higher	8.40 *±* 2.62 [6.13–14.60]	-1.85 *±* 0.98	0	0

The table shows mean *±* SD [range] LCI after reanalysis of the tests with changed apparatus dead space, mean *±* SD change in percentage of standard LCI, number of children with a change between 5–10% and children with >10% change of standard LCI.

### Signal processing and detection limits

**Fig 4:**
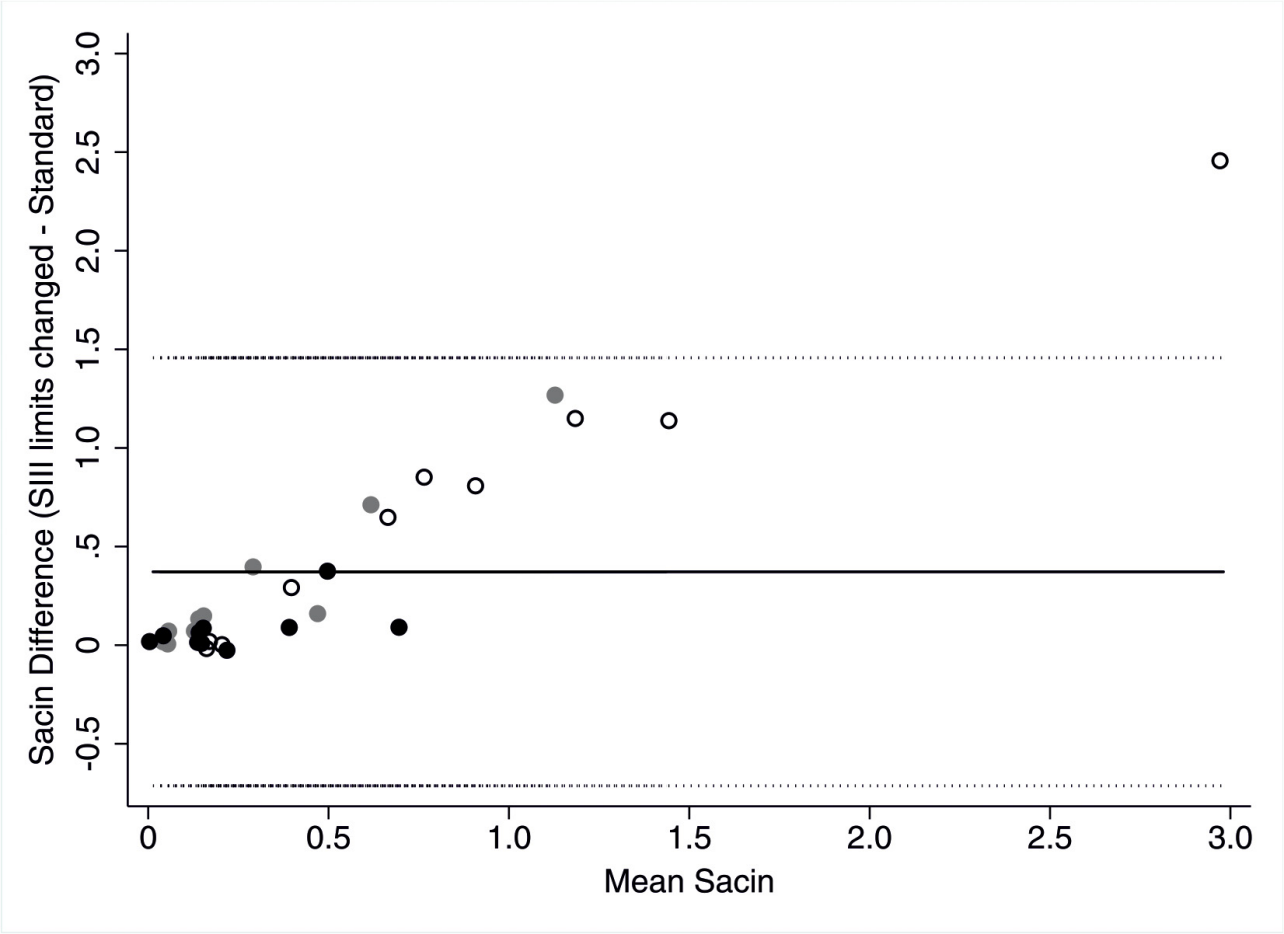
Sacin measurement bias due to changed setting for limits of SIII calculation. Bland Altmann plot of Sacin determined with changed limits for SIII calculation from 65–95% to 50–80% of expired volume minus baseline Sacin versus mean of Sacin. Black circles represent children with CF, grey circles former preterm and open circles healthy children. Solid line represent mean difference (0.372), dotted lines represent upper (1.457) and lower (-0.713) limits of agreement.

Inactivating drift correction of the O_2_ signal marginally increased low LCI and FRC values and decreased high LCI and FRC values which resulted in comparable mean values but showed a clear influence on single measurements (Tables 6 and 7). Inactivation of dynamic CO_2_ correction leads to an overall increase of LCI and FRC without clear systematic effect. For both inactivation of O_2_ drift and dynamic CO_2_ correction 5 children (4 CF, 1 Preterm; and 1CF, 2 Preterm, 2 Healthy) showed >10% LCI change. Inactivation of O_2_ response-time correction lead to a significant increase in LCI with >10% change in 11 children (2 CF, 6 Preterm, 3 Healthy). Thereby measurement bias was weakly linearly and inversely related to FRC magnitude. Inactivating re-inspired N_2_ and increasing the sensitivity of breath volume detection lead to heterogeneous changes of LCI and FRC. Changing limits for SIII calculation from 65–95% to 50–80% of expired volume altered Scond non-systematically, while Sacin significantly increased on average by the double. Sacin showed a linear trend for more increase the higher standard Sacin. This effect was more pronounced in raw Sacin than in volume-corrected Sacin (Fig 4).

**Table 6:** Change of N_2_MBW results after reanalysis with different signal processing and detection limits.

Settings	LCI	FRC	Scond	Scond*VT	Sacin	Sacin*VT
O_2_ drift correction off	↔	↓	↔	(↑)	↔	(↓)
Dynamic CO_2_ correction off	↑↑	↑↑	↓↓	↓	(↑)	↑
O_2_ response-time correction off	↑↑	↓↓	↔	↔	↑↑	↑↑
Integrated reinspired N_2_ off	↔	↔	↔	↔	↔	(↓)
Volume Sensitivity higher	↔	(↓)	(↓)	(↓)	(↑)	↑
SIII 50–80%	↔↔	↔↔	↔	↔	↑↑	↑↑

Change of N_2_MBW test results of 30 children after simulation with changed default algorithms for signal correction. Results of standard N_2_MBW test were compared to the test with changed setting parameter by Wilcoxon signed-rank test and marked as follows: ↓↓ difference of p < 0.001, ↓ p < 0.01, (↓)p < 0.05, ↔ no significant change, ↔↔ no change at all.

**Table 7:** Effect size of the change in LCI after reanalysis with different algorithms for signal processing and detection limits.

Settings	Changed LCI	% change of standard LCI	# children with 5–10% change	# children with >10% change
*Baseline*	*8*.*55 ± 2*.*64* [6.29–14.94]			
O_2_ drift correction off	8.29 *±* 2.18 [6.38–14.95]	-1.75 *±* 7.95	5 (17%)	5 (17%)
Dynamic CO_2_ correction off	9.07 *±* 2.60 [6.65–15.99]	6.67 *±* 5.23	15 (50%)	5 (17%)
Integrated reinspired N_2_ off	8.51 *±* 2.57 [6.25–14.76]	-0.41 *±* 1.65	0	0
O_2_ response-time correction off	9.24 *±* 2.71 [6.63–18.05]	8.65 *±* 6.65	14 (47%)	11 (37%)
Volume Sensitivity higher	8.57 *±* 2.63 [6.25–14.94]	0.23 *±* 0.64	0	0

The table shows mean *±* SD [range] LCI after reanalysis of the tests with changed default algorithms for signal correction, mean *±* SD change in percentage of standard LCI, number of children with a change between 5–10% and children with >10% change of standard LCI.

### Signal delay times

**Fig 5:**
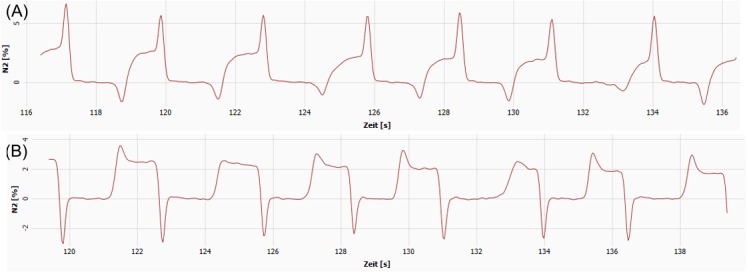
Distortion of the N_2_ signal due to changes of the O_2_ flow-offset setting. (A) Lowering the O_**2**_ flow-offset setting from 618 to 578 ms (-7%) resulted in distorted N_**2**_ signal in the last washout breaths, typically forming spikes at the end of expired signal trace and optionally divots at the end of inspiration. (B) Elevating the O_**2**_ flow-offset setting from 618 to 658 ms (+7%) typically resulted in divots at the beginning of inspiration and optionally spikes at the beginning of expired N_**2**_ signal trace.

Change of O_2_ signal delay time settings had a significant impact on LCI and FRC, starting from changes of ±40 ms upwards (Tables 8 and 9). (i) Decreasing flow O_2_ offset by 40 ms (-7%) lead to a mean LCI increase of 12% and a significant FRC decrease, thereby 60% of the children showed >10% LCI change. The N_2_ signal typically showed spikes at end-expiration over the last washout breaths (Fig 5A). Elevating the flow O_2_ offset by 40 ms (+7%) resulted in the opposite effect with a mean LCI decrease of 9% and 43% of the children with >10% LCI change. This resulted in divots at beginning of inspired N_2_ signal (Fig 5B). Changing flow-CO_2_ offset showed the opposite effect on LCI and FRC but not exceeding 10%, and without visible change of the N_2_ signal. (ii) Combining changes in flow-O_2_ and-CO_2_ offset resulted in the same trend of changes as for flow-O_2_ offset alterations alone. (iii) When maintaining a constant delay difference between the two signals, effect sizes of changes were lower, but still significant. As expected, no effect was visible on the N_2_ signal even in those children with relevant LCI changes. Overall Scond changed non-systematically while Sacin showed the same trend of changes as LCI.

**Table 8:** Change of N_2_MBW results after reanalysis with different signal delay times.

Settings	LCI	FRC	Scond	Scond*VT	Sacin	Sacin*VT
*Individual signal changes*						
O_2_ delay -20ms	↑↑	↓↓	↔	↔	↑↑	↑↑
O_2_ delay -40ms	↑↑	↓↓	↔	↔	↑	↑↑
O_2_ delay -60ms	↑↑	↓↓	↔	↔	↑↑	↑↑
O_2_ delay +20ms	↓↓	↑↑	↔	↔	↓	↓
O_2_ delay +40ms	↓↓	↑↑	(↓)	(↓)	↓↓	↓↓
O_2_ delay +60ms	↓↓	↑↑	↓	↓	↓↓	↓↓
CO_2_ delay -10ms	↓↓	↑↑	(↓)	(↓)	↔	↔
CO_2_ delay +10ms	↑↑	↓↓	↑	↑	↔	↔
*Simultaneous signal changes using different delays*						
O_2_ -100ms and CO_2_ -10ms	↑↑	↓↓	↔	↔	↑↑	↑↑
O_2_ +100ms and CO_2_ +10ms	↓↓	↑↑	↓	↓	↓↓	↓↓
O_2_ -100ms and CO_2_ +10ms	↑↑	↓↓	↔	↔	↑↑	↑↑
O_2_ +100ms and CO_2_ -10ms	↓↓	↑↑	↓↓	↓↓	↓↓	↓↓
*Simultaneous signal changes using similar delays*						
O_2_ -40ms and CO_2_ -40ms	↑↑	↓↓	↔	↔	↑	↑↑
O_2_ +40ms and CO_2_ +40ms	↓↓	↑↑	↔	↔	↓↓	↓↓
O_2_ +80ms and CO_2_ +80ms	↓↓	↑↑	↔	↔	↓↓	↓↓

Change of N_2_MBW test results of 30 children after simulation with changed signal delay times. Results of standard N_2_MBW test were compared to the test with changed setting parameter by Wilcoxon signed-rank test and marked as follows: ↓↓ difference of p < 0.001, ↓ p < 0.01, (↓) p < 0.05, ↔ no significant change. Of note, for simultaneous signal changes using similar time steps, delays between O_2_ and CO_2_ sensor were kept constant.

**Table 9:** Effect size of the change in LCI after reanalysis with different signal delay times.

Settings	Changed LCI	% change of standard LCI	# children with 5–10% change	# children with>10% change
*Baseline*	*8*.*55 ± 2*.*64* [6.29–14.94]			
*Individual signal changes*				
O_2_ delay -20ms	9.03 ± 2.79 [6.55–16.82]	5.68 ± 3.85	16 (53%)	3 (10%)
O_2_ delay -40ms	9.53 ± 2.86 [6.69–19.20]	11.98 ± 8.21	7 (23%)	18 (60%)
O_2_ delay -60ms	10.33 ± 3.20 [6.83–21.30]	21.37 ±12.93	4 (13%)	25 (83%)
O_2_ delay +20ms	8.13 ± 2.46 [5.93–13.54]	-4.87 ± 2.33	13 (43%)	1 (3%)
O_2_ delay +40ms	7.76 ± 2.35 [5.55–12.85]	-9.12 ± 3.46	15 (50%)	13 (43%)
O_2_ delay +60ms	7.47 ± 2.22 [5.27–12.36]	-12.47 ± 4.71	7 (23%)	21 (70%)
CO_2_ delay -10ms	8.48 *±* 2.60 [6.23–14.50]	-0.86 *±* 1.22	0	0
CO_2_ delay +10ms	8.62 *±* 2.69 [6.32–15.25]	0.65 *±* 1.06	0	0
*Simultaneous signal changes using different delays*				
O_2_ -100ms, CO_2_ -10ms	13.88 *±* 6.84 [7.23–40.43]	61.48 *±*54.85	0	30 (100%)
O_2_ +100ms, CO_2_ +10ms	7.14 *±* 2.20 [5.04–12.03]	-16.38 *±* 6.21	6 (20%)	24 (80%)
O_2_ -100ms, CO_2_ +10ms	14.96 *±* 8.61 [7.30–46.88]	73.89 *±*79.56	0	30 (100%)
O_2_ +100ms, CO_2_ -10ms	7.04 *±* 2.16 [4.98–11.81]	-17.57 *±* 6.33	4 (13%)	26 (87%)
*Simultaneous signal changes using similar delays*				
O_2_ -40ms, CO_2_ -40ms	9.28 *±* 2.81 [6.61–17.29]	8.79 *±* 6.74	9 (30%)	14 (47%)
O_2_ +40ms, CO_2_ +40ms	7.95 *±* 2.47 [5.72–13.49]	-7.12 *±* 3.36	18 (60%)	6 (20%)
O_2_ +80ms, CO_2_ +80ms	7.49 *±* 2.36 [5.33–12.69]	-12.56 *±* 4.68	8 (27%)	21 (70%)

The table shows mean *±* SD [range] LCI after reanalysis of the tests with changed signal delay times, mean ± SD change in percentage of standard LCI, number of children with a change between 5–10% and children with >10% change of standard LCI. Of note, for simultaneous signal changes using similar time steps, delays between O_2_ and CO_2_ sensor were kept constant.

Taken together, signal delay times had the largest impact upon the primary outcomes LCI (Table 10), FRC, and secondary outcomes Scond and Sacin. Thereby changes in O_2_ delay were relatively more sensitive than changes in CO_2_ delay; while 7% (40 ms) change in O_2_ delay showed a significant impact, 17–25% (10 ms) change in CO_2_ delay had no significant effect. The largest effect on Sacin was caused by changed limits for SIII calculation doubling Sacin values on average. Except for inactivation of O_2_ drift or reinspired N_2_ leading to different changes among disease groups, changes of software factors lead to the same trend of LCI and FRC changes in all children independent of disease state. This uniformity often generated an overall statistically significant difference which was not necessarily clinically relevant (LCI change >10%) in all subjects (Table 10).

**Table 10:** Summary of the impact of different software settings on LCI of N_2_MBW.

LCI change	Statistical significance	Clinical relevance	Max. No. of children with>10% change
Environmental conditions	Yes	No	2 (7%)
Apparatus dead space	Yes	No	0
Signal processing and detection limits	Only for algorithms concerning O_2_ and CO_2_ signal processing	Yes, for those mentioned	11 (37%)
Signal delay times	Yes, for O_2_ delay changes of ±40 ms upwards	Yes	18 (60%)

Overview of impact on LCI after change of software settings. High quality N_2_MBW tests of 30 children with different lung disease were reanalyzed with changed software settings. Several parameters were changed according to four sectors, environmental conditions (e.g. ambient temperature), setup parameter (apparatus dead space), software algorithm (e.g. O_2_ response-time correction), and flow-gas signal delay times. Because of the uniformity of change statistical significance might be reached over the study population without clinical relevance (defined as >10% change of baseline LCI for the individual measurement; and for the change in software setting if >10% of all children showed such a LCI change >10%).

## Discussion

This is the first study that systematically examines the implication of different software settings on washout results in a commercially available N_2_MBW software. We find that incorrect software settings introduce significant measurement bias with different effect sizes of various settings. While some settings (e.g. environmental settings) introduce only small random measurement errors, other software settings show significant impact upon results. The most susceptible settings were signal alignments, realistic changes in flow-O_2_ signal delay beyond ±40 ms resulted in >10% LCI change in 60% of the children for -40 ms O_2_ change and in 43% of the children for +40 ms O_2_ change. Incorrect apparatus dead space estimates may especially bias smaller FRC measurements in young children. While some of the current findings are specific for this setup, principles are also valid for other MBW devices currently available. Especially, signal alignment is done in all MBW setups.

The current data draws attention to important software settings which have not been reported in detail before. Various efforts have been made for validation of available washout setups using a realistic lung model for FRC measurement [13;17;18] or by comparison to mass spectrometry [19–22] using optimized software settings. While the ERS/ATS consensus statement gives recommendations for the optimal hard- and software there is only little data at what point technical factors lead to relevant measurement errors [11;12].

There is preliminary evidence that change of settings over time in two different software releases of the same software lead to different FRC and LCI results as seen in infant MBW using sulfur hexafluoride (SF_6_) [23]. Two other studies simulated specifically flow gas misalignment in different washout setups, both using 10 ms steps from -50 ms to +50 ms [24;25]. While Horsley et al. simulated flow-gas misalignment in a lung model using SF_6_ washouts with an Innocor gas analyzer (Innovision, Odense, Denmark), Buess et al. analyzed raw data files from N_2_MBW (ndd Medical Technologies, Switzerland) [24;25]. Both found an almost linear increase of FRC between ±2.5% and ±7.5% over the -50 to +50 ms delay-time change and a higher FRC error with higher breathing frequency. While different setups and simulation conditions hamper direct translation of results, direction and effect size seem comparable to FRC errors in our study. In any case, all those studies point towards the importance of precise flow and gas signal alignment on lung volume calculations particularly in young children with faster ventilation. This has now been shown for all commercially available MBW setups.

One limitation of our study is that we simulated technical measurement errors rather than having the subjects themselves performing the test repetitively under changed conditions. Change of certain settings might have a different impact on real-time washout measurement compared to simulated tests, e.g. the change of post-capillary dead space would be associated with a change of signal delay times concurrently. On the other hand we based our simulations on the reload of raw, unprocessed storage files of the original measurement. Thus we are confident that results reflect real-life impact. To enable multiple reloads of the tests, we used averaged delay times per set. As we did not change the setup, individual delay times of the tests varied only minimally within a narrow range (Table 1). In addition we confirmed proper flow-gas alignment for each test by visual control of the N_2_ signal shape. Thus, we believe this approach does not impede validity of results. Another limitation is that results are specific for the device used in our study. However only certain findings do not apply to other pieces of equipment, like the change of software specific algorithms. General findings such as for signal alignement and BTPS correction are not specific to this apparatus. Sampling flow and gas concentration at different points will always result in delay between signals. Most of the times this delay is even flow-dependent [26]. This applies to all available and customized washout setups such as ultrasonic flowmeter based MBW using either N_2_ [14] or SF_6_ [27], Innocor gas analyser using SF_6_ [18] and mass spectrometer using SF_6_ [28]. As mentioned above this is underlined by comparable findings of incorrect flow-gas delays on MBW results also for other devices [18;24;25].

We only tested single relevant changes for each software factor within our heterogeneous study population. Thus we could not assess the effect over the complete range of the factor, or define clear relationships with outcome parameters. The Bland-Altman plots suggest nonlinearity for many parameters. However the primary aim of our study was to tease out the most important software setting by including measurement of good quality [10] in a wide age range of children with different lung disease. The majority of our findings were consistent throughout the study population resulting in the same trend of change for all children. Depending on the role of software settings within the algorithm for outcome calculation, the effects of changes of software settings affecting primarily volume measurements were independent of underlying LCI (e.g. BTPS, temperature) while the effect of changes in settings primarily affecting gas concentrations (e.g. reinspired N_2_) was of course related to underlying LCI (e.g. reinspired N_2_).

The most relevant finding was the significant impact of flow-O_2_ signal misalignment on FRC and LCI results. The same applied for Scond and Sacin, with >10% change of their baseline values. The O_2_ signal mainly determines the tracer gas signal (N_2_) in the current setup. In order to derive FRC from MBW measurements, expired gas volumes are calculated by integrating the gas signal with flow. The same applies for measurement of respiratory dead space [26;29]. This requires precise alignment of gas and flow signals [30]. In the N_2_MBW setup we encounter two additional challenges for this alignment. During N_2_ washout gas composition changes due to increasing O_2_ concentration and viscosity [30]. This leads to changing delay times even in the side-stream sampling of O_2_ over the period of the washout. Moreover mainstream flow is not constant because of breathing cycles. This will influence signal alignment furthermore. So far, flow gas delay was calculated as fixed correction factor for the whole measurement. The susceptibility of this flow gas synchronization and the clinical implication in case of misalignment as shown in this study suggests strongly that this algorithm is prone to errors. Whether a dynamic flow-gas delay correction is superior, especially at younger age, in which these errors are more relevant [24;25], needs to be examined in future studies. Simulation of changes in equipment dead space also showed more impact in young children, where dead space is large relative to lung volumes. This is in line with recently published clinical data showing higher LCI values with increasing equipment dead space especially in young children [31].

Technical quality control of washout tests gets particularly important for longitudinal studies with potentially changing software settings. In the current setup, different signal processing settings (such as O_2_-drift correction, dynamic CO_2_ correction or correction for re-inspired N_2_ volume) were implemented continuously over three years. Moreover the storage of unprocessed raw data makes it possible to rerun original measurement with changed settings. We now know that the most important technical factor to check for the operator is correct signal alignment throughout the entire washout while other factors such as small deviations from environmental settings or BTPS correction only have small and clinically negligible impact. Unfortunately, if the N_2_ signal shows relevant flow-gas misalignment, it gets clearly visible to the operator only over the last washout breaths. Prior to software updates the companies should provide data showing the new software’s impact (if any) on MBW outcomes. This is recommended by the current consensus [10] and would enable the user to account for possible measurement bias, which seems especially important for repeated MBW measurements in longitudinal studies.

To sum up, we found that software settings have clear impacts upon MBW results. The most important technical factor is flow gas signal alignment. Particularly inaccurate flow-O_2_ offset in N_2_MBW can lead to wrongly elevated or false normal LCI in a wide age range of children with different lung disease. Whether a flow-adapted new algorithm for signal synchronization will lead to more robust results needs to be examined in the future.

## Supporting Information

S1 Minimal Dataset In this dataset the individual settings and respective outcomes are given for all changes in the software settings as detailed in the manuscript. (TXT)
